# Editorial: Crohn's disease: nutritional strategies to improve patient's quality of life

**DOI:** 10.3389/fnut.2024.1425859

**Published:** 2024-06-19

**Authors:** Iolanda Cioffi, Lidia Santarpia

**Affiliations:** ^1^Division of Human Nutrition, Department of Food, Environmental and Nutritional Sciences—DEFENS, Università degli Studi di Milano, Milan, Italy; ^2^Department of Clinical Medicine and Surgery, Federico II University Hospital, Naples, Italy

**Keywords:** malnutrition, inflammatory bowel disease, diet, nutraceutical, body composition

Crohn's disease (CD) is a chronic relapsing, inflammatory disorder of the gastrointestinal tract characterized by an unpredictable course. Systemic inflammation together with disease symptoms such as malabsorption, abdominal pain, chronic diarrhea, and reduced appetite can remarkably increase the risk of developing malnutrition in patients with CD, negatively impacting on the quality of life. Also, it is widely recognized that poor nutritional status is a predictive factor that worsens clinical outcome in this pathology. Therefore, an early diagnosis of malnutrition and/or nutritional risk along with an effective nutritional management, supported by reliable tools, can play a pivotal role in prolonging remission and decreasing the incidence of post-operative complications.

This Research Topic delves into recent research that provides insight into many facets of nutritional evaluation and control in CD. This Research Topic consists of four primary research articles (Gravina et al.; Huang et al.; Cioffi et al.; Tang et al.) and a narrative review (Alves Martins et al.), which together offer unique insights on the role of diet in the management of CD.

Three out four articles covered different aspects of nutritional assessment, while the fourth concerned the use of supplementation, in patients with inflammatory bowel disease (IBD). Gravina et al. described the results from an *ex vivo* experimental model testing the anti-inflammatory effects of a nutraceutical compound on the intestinal mucosa obtained from naïve IBD patients. Incubation with a mixture of berberine, quercetin, and *Hericium erinaceus* in combination with vitamin B3 and B8, reduced the expression of proinflammatory cytokines at both mRNA and protein levels in the tissues of these patients. However, these promising results may warrant further investigations to confirm any beneficial effects.

Since malnutrition is often observed in CD patients and is generally correlated with the severity of the disease, the original article by Huang et al. evaluated the reliability of the Global Leadership Initiative on Malnutrition (GLIM) criteria in these patients and aimed to provide a faster tool for the diagnosis of malnutrition. The authors found that GLIM criteria were able to identify malnourished patients and that the use of the Harvey-Bradshaw index (HBI) score could be considered as an additional etiologic criterion of inflammation. Furthermore, the development of the HBM nomogram, incorporating three independent predictors of malnutrition, exhibited a powerful discriminatory ability and reliable calibration performance.

Among nutrients, amino acids play a key role in the maintenance of intestinal and immune homeostasis, by modulating gut inflammation in IBD. A fascinating scenario on a possible correlation between serum amino acid (AA) profiles and disease activity in patients with CD has been investigated by Cioffi et al. in an exploratory analysis performed in a large cohort of patients. The authors showed that serum levels of some AAs such as lysine, leucine, valine, and glutamine were lower in patients with active CD compared to those clinically quiescent. On the contrary, aspartic and glutamic acid were higher in active patients and directly associated with disease activity and interleukin-1β levels. In addition, other correlations were found between serum essential AAs and dietary protein intake. These results could encourage future investigations concerning the relationship between specific AAs, disease activity and diet in patients with CD.

In CD patients treated with infliximab, Tang et al. showed a correlation between body composition parameters, calculated at the 3rd lumbar vertebra using computed tomography (CT), and disease severity endoscopically evaluated. They found that body composition parameters changed among patients with different levels of disease activity. In particular, subcutaneous adiposity index (SAI) and skeletal mass index (SMI) were positively associated with disease severity, while visceral adiposity index (VAI) was negatively correlated; of these, SMI gave the best performance.

Lastly, the narrative review by Alves Martins et al. underlined the knowledge gap of how nutritional deficiency can impact on perianal disease, which has a poor prognosis and a disabling course. Although lacking evidence, the incidence of nutritional disorders in patients with perianal CD seemed significantly high, with potential implications on clinical outcomes and quality of life. Hence, a tailored nutritional assessment is crucial to avoid malnutrition and improve clinical outcomes in CD patients with perianal forms.

All together, these studies provided valuable understandings on how the assessment of nutritional status as well as of potential nutraceutical approach could influence disease activity, quality of life and clinical outcomes in patients with IBD. Indeed, a multidimensional nutritional evaluation should consider the use of screening tools followed by the analysis of body composition, dietary intake, and potential new diet-related biomarkers. Also, the importance of disease activity and complications linked to both phenotype and disease location has been acknowledged. Therefore, a greater attention should be paid on nutrition in future research involving patients with IBD, by promptly identifying those who would benefit of a targeted nutritional approach, either as a standalone therapy or in combination with medical treatments, to achieve remission and improve disease outcomes.

## Author contributions

IC: Writing – original draft, Writing – review & editing. LS: Writing – original draft, Writing – review & editing.

